# [(*E*)-(1-Phenyl­ethyl­idene)amino]­urea methanol monosolvate

**DOI:** 10.1107/S1600536811000225

**Published:** 2011-01-12

**Authors:** Guang-Bin Liu, Peng-Sheng Chen, Chang-Xiang Liu, Ling Fu, Xu-Liang Nie

**Affiliations:** a Department of Chemistry, Jiangxi Agricultural University, Nanchang 330045, People’s Republic of China; b Guanlian Middle School of Wuning, Jiangxi Wuning 332302, People’s Republic of China

## Abstract

In the title compound, C_9_H_11_N_3_O·CH_4_O, the semicarbazone moiety is nearly planar [maximum deviation = 0.017 (2) Å] and is twisted by a dihedral angle of 29.40 (13)° with respect to the phenyl ring. The semicarbazone moiety and phenyl ring are located on opposite sides of the C=N bond, showing the *E* configuration. An inter­molecular O—H⋯O and N—H⋯O hydrogen-bonding network occurs in the crystal structure.

## Related literature

For general background and applications of semicarbazone derivatives, see: Chandra & Gupta (2005 ▶). For related structures, see: Fun *et al.* (2009*a* ▶,*b* ▶).
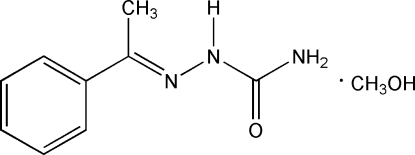


## Experimental

### 

#### Crystal data

C_9_H_11_N_3_O·CH_4_O
*M* *_r_* = 209.25
Monoclinic, 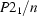

*a* = 6.629 (3) Å
*b* = 8.371 (4) Å
*c* = 20.329 (9) Å
β = 99.181 (5)°
*V* = 1113.6 (8) Å^3^
*Z* = 4
Mo *K*α radiation
μ = 0.09 mm^−1^
*T* = 296 K
0.24 × 0.22 × 0.18 mm

#### Data collection

Bruker APEXII CCD diffractometer
8148 measured reflections
2057 independent reflections
1617 reflections with *I* > 2σ(*I*)
*R*_int_ = 0.027

#### Refinement

*R*[*F* ^2^ > 2σ(*F* ^2^)] = 0.044
*wR*(*F* ^2^) = 0.128
*S* = 1.07
2057 reflections
140 parameters
H-atom parameters constrained
Δρ_max_ = 0.26 e Å^−3^
Δρ_min_ = −0.18 e Å^−3^

### 

Data collection: *APEX2* (Bruker, 2007 ▶); cell refinement: *SAINT* (Bruker, 2007 ▶); data reduction: *SAINT*; program(s) used to solve structure: *SHELXTL* (Sheldrick, 2008 ▶); program(s) used to refine structure: *SHELXTL*; molecular graphics: *SHELXTL*; software used to prepare material for publication: *SHELXTL*.

## Supplementary Material

Crystal structure: contains datablocks global, I. DOI: 10.1107/S1600536811000225/xu5132sup1.cif

Structure factors: contains datablocks I. DOI: 10.1107/S1600536811000225/xu5132Isup2.hkl

Additional supplementary materials:  crystallographic information; 3D view; checkCIF report

## Figures and Tables

**Table 1:** Hydrogen-bond geometry (Å, °)

*D*—H⋯*A*	*D*—H	H⋯*A*	*D*⋯*A*	*D*—H⋯*A*
O2—H2*A*⋯O1^i^	0.82	1.93	2.745 (2)	177
N2—H8⋯O1^ii^	0.86	2.10	2.936 (2)	164
N3—H3*A*⋯O2^iii^	0.86	2.12	2.953 (2)	164
N3—H3*B*⋯O2	0.86	2.36	3.042 (2)	137

Symmetry codes: (i) 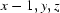; (ii) 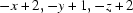; (iii) 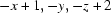.

**Fig. 1.**
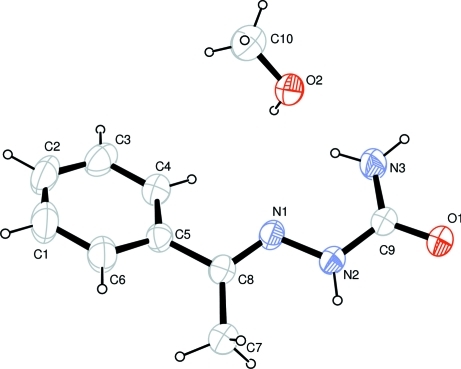
The molecular structure of (I), showing the atom-labelling scheme and displacement ellipsoids at the 50% probability level.

**Fig. 2.**
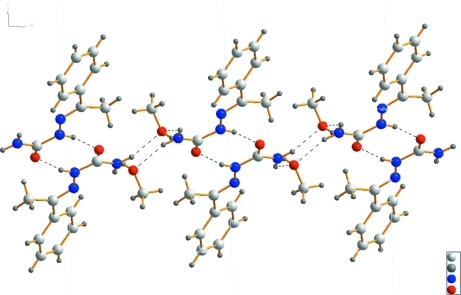
The crystal packing of (I), showing a one-dimensional chain down the b axis; H-bonds are shown as dashed lines.

**Fig. 3.**
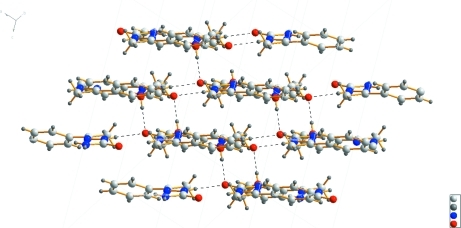
The crystal packing of (I), showing a two-dimensional sheet; H-bonds are shown as dashed lines.

## supplementary crystallographic information

### Comment

The semicarbazone is a derivative of an aldehyde or ketone formed by a condensation between a ketone or aldehyde and semicarbazide. It is widely used in field of organometalics (Chandra & Gupta, 2005). Several crystal structures have recently reported by Fun *et al.*, 2009a,b. Here we report the crystal structure of the title compound, (I).

In (I) (Fig. 1), the semicarbazone group is nearly planar, with the maximum deviation of 0.017 (2) Å. The mean plane of semicarbazone group and the benzene ring makes a dihedral angle of 29.40 (13)°. In the crystal structure there is also a methanol molecular which is stabilized by N—H···O hydrogen bond with the semicarbazone group. The methanol molecular further linked the semicarbazone group adjacent into a one-dimensional chain by N—H···O hydrogen bonds formed along the *b* axis. These chains are further linked *via* pairs of O—H···O hydrogen bonds involving the methanol O atoms and semicarbazone O atoms to a two-dimensional hydrogen bonds framework (Fig. 3).

### Experimental

Semicarbazide hydrochloride (11 g, 0.1 mol) was dissolved in water (100 ml), and sodium acetate (16.4 g, 0.2 mol) was added and dissolved by stirring at room temperature. To this, acetophenone (11.4 g, 0.095 mol) in ethanol (60 ml) was then added, and the mixture stirred well for 2 h at 323 K using a modified Vilsmeier-Haak reaction. The separated crystals were filtered, washed with cold water and recrystallized from methanol solution.

### Refinement

All H atoms were included in calculated positions and refined as riding atoms, with C—H = 0.93–0.96, O—H = 0.82 and N—H = 0.86 Å, with *U*_iso_(H) = 1.5 *U*_eq_(C,O) for methyl and hydroxyl H atoms and 1.2*U*_eq_(C,N) for the others.

### Crystal data

**Table:**

C_9_H_11_N_3_O·CH_4_O	*F*(000) = 448
*M**_r_* = 209.25	*D*_x_ = 1.248 Mg m^−^^3^
Monoclinic, *P*2_1_/*n*	Mo *K*α radiation, λ = 0.71073 Å
Hall symbol: -P 2yn	Cell parameters from 2808 reflections
*a* = 6.629 (3) Å	θ = 2.6–28.2°
*b* = 8.371 (4) Å	µ = 0.09 mm^−^^1^
*c* = 20.329 (9) Å	*T* = 296 K
β = 99.181 (5)°	Block, colourless
*V* = 1113.6 (8) Å^3^	0.24 × 0.22 × 0.18 mm
*Z* = 4	

### Data collection

**Table:**

Bruker APEXII CCD diffractometer	1617 reflections with *I* > 2σ(*I*)
Radiation source: fine-focus sealed tube	*R*_int_ = 0.027
graphite	θ_max_ = 25.5°, θ_min_ = 2.6°
φ and ω scans	*h* = −8→8
8148 measured reflections	*k* = −9→10
2057 independent reflections	*l* = −24→24

### Refinement

**Table:**

Refinement on *F*^2^	Secondary atom site location: difference Fourier map
Least-squares matrix: full	Hydrogen site location: inferred from neighbouring sites
*R*[*F*^2^ > 2σ(*F*^2^)] = 0.044	H-atom parameters constrained
*wR*(*F*^2^) = 0.128	*w* = 1/[σ^2^(*F*_o_^2^) + (0.0583*P*)^2^ + 0.3389*P*] where *P* = (*F*_o_^2^ + 2*F*_c_^2^)/3
*S* = 1.07	(Δ/σ)_max_ = 0.001
2057 reflections	Δρ_max_ = 0.26 e Å^−^^3^
140 parameters	Δρ_min_ = −0.18 e Å^−^^3^
0 restraints	Extinction correction: *SHELXTL* (Sheldrick, 2008), Fc^*^=kFc[1+0.001xFc^2^λ^3^/sin(2θ)]^-1/4^
Primary atom site location: structure-invariant direct methods	Extinction coefficient: 0.020 (4)

### Special details

**Table:**

Geometry. All e.s.d.'s (except the e.s.d. in the dihedral angle between two l.s. planes) are estimated using the full covariance matrix. The cell e.s.d.'s are taken into account individually in the estimation of e.s.d.'s in distances, angles and torsion angles; correlations between e.s.d.'s in cell parameters are only used when they are defined by crystal symmetry. An approximate (isotropic) treatment of cell e.s.d.'s is used for estimating e.s.d.'s involving l.s. planes.
Refinement. Refinement of *F*^2^ against ALL reflections. The weighted *R*-factor *wR* and goodness of fit *S* are based on *F*^2^, conventional *R*-factors *R* are based on *F*, with *F* set to zero for negative *F*^2^. The threshold expression of *F*^2^ > σ(*F*^2^) is used only for calculating *R*-factors(gt) *etc*. and is not relevant to the choice of reflections for refinement. *R*-factors based on *F*^2^ are statistically about twice as large as those based on *F*, and *R*- factors based on ALL data will be even larger.

### Fractional atomic coordinates and isotropic or equivalent isotropic displacement parameters (Å^2^)

**Table:**

	*x*	*y*	*z*	*U*_iso_*/*U*_eq_	
C1	0.0487 (3)	0.6825 (3)	0.73837 (11)	0.0636 (6)	
H1	0.0233	0.7479	0.7010	0.076*	
C2	−0.0980 (3)	0.5772 (3)	0.75182 (11)	0.0630 (6)	
H2	−0.2233	0.5717	0.7238	0.076*	
C3	−0.0590 (3)	0.4793 (3)	0.80720 (10)	0.0550 (6)	
H3	−0.1582	0.4076	0.8163	0.066*	
C4	0.1262 (3)	0.4875 (2)	0.84901 (9)	0.0438 (5)	
H4	0.1504	0.4216	0.8863	0.053*	
C5	0.2773 (2)	0.5931 (2)	0.83614 (8)	0.0371 (4)	
C6	0.2342 (3)	0.6916 (2)	0.78033 (10)	0.0521 (5)	
H6	0.3319	0.7645	0.7712	0.063*	
C7	0.6059 (3)	0.7470 (2)	0.88452 (10)	0.0509 (5)	
H7A	0.6457	0.7752	0.9305	0.076*	
H7B	0.5268	0.8322	0.8617	0.076*	
H7C	0.7257	0.7294	0.8645	0.076*	
C8	0.4800 (2)	0.5973 (2)	0.87964 (8)	0.0362 (4)	
C9	0.7858 (2)	0.3143 (2)	0.97715 (8)	0.0361 (4)	
C10	0.0959 (4)	0.0359 (3)	0.89062 (11)	0.0673 (6)	
H10A	−0.0277	0.0967	0.8798	0.101*	
H10B	0.0627	−0.0746	0.8957	0.101*	
H10C	0.1762	0.0466	0.8555	0.101*	
N1	0.5332 (2)	0.46585 (17)	0.91022 (7)	0.0362 (4)	
N2	0.7209 (2)	0.45770 (17)	0.95027 (7)	0.0398 (4)	
H8	0.7957	0.5416	0.9582	0.048*	
O1	0.96431 (17)	0.30074 (14)	1.00688 (6)	0.0458 (4)	
N3	0.6552 (2)	0.19317 (18)	0.96944 (9)	0.0526 (5)	
H3A	0.6927	0.1007	0.9853	0.063*	
H3B	0.5330	0.2071	0.9486	0.063*	
O2	0.20857 (19)	0.09309 (17)	0.95077 (7)	0.0544 (4)	
H2A	0.1370	0.1538	0.9688	0.082*	

### Atomic displacement parameters (Å^2^)

**Table:**

	*U*^11^	*U*^22^	*U*^33^	*U*^12^	*U*^13^	*U*^23^
C1	0.0641 (14)	0.0727 (15)	0.0482 (12)	0.0140 (12)	−0.0092 (10)	0.0110 (11)
C2	0.0449 (11)	0.0791 (16)	0.0569 (13)	0.0150 (11)	−0.0162 (10)	−0.0122 (12)
C3	0.0361 (10)	0.0651 (13)	0.0613 (13)	−0.0020 (9)	0.0001 (9)	−0.0118 (11)
C4	0.0392 (9)	0.0484 (11)	0.0427 (10)	0.0015 (8)	0.0032 (8)	0.0004 (8)
C5	0.0372 (9)	0.0385 (9)	0.0344 (9)	0.0060 (7)	0.0021 (7)	−0.0030 (7)
C6	0.0502 (11)	0.0547 (12)	0.0484 (11)	0.0029 (9)	−0.0009 (9)	0.0092 (9)
C7	0.0467 (10)	0.0442 (11)	0.0569 (12)	−0.0038 (8)	−0.0063 (9)	0.0047 (9)
C8	0.0360 (9)	0.0400 (10)	0.0319 (9)	0.0005 (7)	0.0030 (7)	0.0003 (7)
C9	0.0318 (8)	0.0383 (9)	0.0365 (9)	−0.0005 (7)	0.0008 (7)	0.0000 (7)
C10	0.0706 (14)	0.0675 (15)	0.0605 (14)	−0.0034 (12)	0.0005 (12)	−0.0025 (11)
N1	0.0310 (7)	0.0409 (8)	0.0346 (8)	−0.0004 (6)	−0.0012 (6)	0.0024 (6)
N2	0.0334 (7)	0.0370 (8)	0.0450 (9)	−0.0043 (6)	−0.0058 (6)	0.0042 (6)
O1	0.0337 (7)	0.0422 (7)	0.0562 (8)	0.0000 (5)	−0.0085 (6)	0.0011 (6)
N3	0.0356 (8)	0.0401 (9)	0.0758 (12)	−0.0042 (7)	−0.0096 (8)	0.0125 (8)
O2	0.0420 (7)	0.0510 (9)	0.0658 (9)	0.0044 (6)	−0.0047 (6)	−0.0037 (7)

### Geometric parameters (Å, °)

**Table:**

C1—C2	1.372 (3)	C7—H7C	0.9600
C1—C6	1.382 (3)	C8—N1	1.285 (2)
C1—H1	0.9300	C9—O1	1.2453 (19)
C2—C3	1.383 (3)	C9—N3	1.326 (2)
C2—H2	0.9300	C9—N2	1.360 (2)
C3—C4	1.379 (2)	C10—O2	1.411 (2)
C3—H3	0.9300	C10—H10A	0.9600
C4—C5	1.391 (3)	C10—H10B	0.9600
C4—H4	0.9300	C10—H10C	0.9600
C5—C6	1.395 (3)	N1—N2	1.3757 (18)
C5—C8	1.486 (2)	N2—H8	0.8600
C6—H6	0.9300	N3—H3A	0.8600
C7—C8	1.500 (3)	N3—H3B	0.8600
C7—H7A	0.9600	O2—H2A	0.8200
C7—H7B	0.9600		
			
C2—C1—C6	120.1 (2)	H7A—C7—H7C	109.5
C2—C1—H1	120.0	H7B—C7—H7C	109.5
C6—C1—H1	120.0	N1—C8—C5	114.83 (15)
C1—C2—C3	119.75 (18)	N1—C8—C7	125.18 (15)
C1—C2—H2	120.1	C5—C8—C7	119.98 (15)
C3—C2—H2	120.1	O1—C9—N3	122.64 (15)
C4—C3—C2	120.3 (2)	O1—C9—N2	119.38 (15)
C4—C3—H3	119.8	N3—C9—N2	117.96 (14)
C2—C3—H3	119.8	O2—C10—H10A	109.5
C3—C4—C5	120.83 (18)	O2—C10—H10B	109.5
C3—C4—H4	119.6	H10A—C10—H10B	109.5
C5—C4—H4	119.6	O2—C10—H10C	109.5
C4—C5—C6	117.92 (16)	H10A—C10—H10C	109.5
C4—C5—C8	120.83 (15)	H10B—C10—H10C	109.5
C6—C5—C8	121.22 (16)	C8—N1—N2	118.77 (14)
C1—C6—C5	121.1 (2)	C9—N2—N1	118.67 (13)
C1—C6—H6	119.5	C9—N2—H8	120.7
C5—C6—H6	119.5	N1—N2—H8	120.7
C8—C7—H7A	109.5	C9—N3—H3A	120.0
C8—C7—H7B	109.5	C9—N3—H3B	120.0
H7A—C7—H7B	109.5	H3A—N3—H3B	120.0
C8—C7—H7C	109.5	C10—O2—H2A	109.5
			
C6—C1—C2—C3	−0.5 (3)	C6—C5—C8—N1	−152.23 (17)
C1—C2—C3—C4	0.2 (3)	C4—C5—C8—C7	−154.55 (18)
C2—C3—C4—C5	−0.4 (3)	C6—C5—C8—C7	27.1 (3)
C3—C4—C5—C6	0.9 (3)	C5—C8—N1—N2	178.39 (14)
C3—C4—C5—C8	−177.47 (17)	C7—C8—N1—N2	−0.9 (3)
C2—C1—C6—C5	1.0 (3)	O1—C9—N2—N1	171.57 (15)
C4—C5—C6—C1	−1.2 (3)	N3—C9—N2—N1	−7.4 (2)
C8—C5—C6—C1	177.18 (18)	C8—N1—N2—C9	−173.38 (16)
C4—C5—C8—N1	26.1 (2)		

### Hydrogen-bond geometry (Å, °)

**Table:**

*D*—H···*A*	*D*—H	H···*A*	*D*···*A*	*D*—H···*A*
O2—H2A···O1^i^	0.82	1.93	2.745 (2)	177
N2—H8···O1^ii^	0.86	2.10	2.936 (2)	164
N3—H3A···O2^iii^	0.86	2.12	2.953 (2)	164
N3—H3B···O2	0.86	2.36	3.042 (2)	137

Symmetry codes: (i) *x*−1, *y*, *z*; (ii) −*x*+2, −*y*+1, −*z*+2; (iii) −*x*+1, −*y*, −*z*+2.
